# Image-guided moderately hypofractionated radiotherapy for localized prostate cancer: a multicentric retrospective study (IPOPROMISE)

**DOI:** 10.1007/s11547-024-01782-2

**Published:** 2024-02-19

**Authors:** Gianluca Ingrosso, Elisabetta Ponti, Giulio Francolini, Saverio Caini, Simona Fondelli, Roberto Santini, Maurizio Valeriani, Luciana Rago, Giacomo Duroni, Alessio Bruni, Antonietta Augurio, Francesco Tramacere, Fabio Trippa, Donatella Russo, Marta Bottero, Maria Tamburo, Silvana Parisi, Simona Borghesi, Andrea Lancia, Sara Gomellini, Silvia Scoccianti, Marco Stefanacci, Gianluca Vullo, Teodora Statuto, Giulia Miranda, Bianca Santo, Alessandro Di Marzo, Rita Bellavita, Annamaria Vinciguerra, Lorenzo Livi, Cynthia Aristei, Niccolò Bertini, Carolina Orsatti, Beatrice Detti

**Affiliations:** 1 Radiation Oncology Section, Department of Medicine and Surgery, University of Perugia and Perugia General Hospital, Perugia, Italy; 2 Radiation Oncology Department, San Giovanni Addolorata Hospital, Rome, Italy; 3 Radiation Oncology Unit, Azienda Ospedaliero-Universitaria Careggi, Florence, Italy; 4 Cancer Risk Factors and Lifestyle Epidemiology Unit, Institute for Cancer Research, Prevention and Clinical Network (ISPRO), 50139 Florence, Italy; 5 Radiation Oncology Unit, Department of Oncology, Santa Maria Annunziata Hospital, Azienda USL Toscana Centro, Florence, Italy; 6 Department of Radiation Oncology, Ospedale San Jacopo Pistoia, Pistoia, Italy; 7 Radiotherapy Oncology, Department of Medicine and Surgery and Translational Medicine, Sapienza University of Rome, S. Andrea Hospital, Rome, Italy; 8 Radiation Oncology Unit, IRCCS -CROB, Rionero in Vulture, Potenza Italy; 9 Clinical Epidemiology Unit, Institute for Cancer Research, Prevention and Clinical Network (ISPRO), 50139 Florence, Italy; 10 Radiation Therapy Unit, Department of Oncology and Hematology, University Hospital of Modena, Modena, Italy; 11 Department of Radiation Oncology, “SS Annunziata” Hospital, “G. D’Annunzio” University, Chieti, Italy; 12 Department of Radiation Oncology, Azienda Sanitaria Locale, 72100 Brindisi, Italy; 13 Department of Radiotherapy, Saint Maria Hospital, Terni, Italy; 14 Radiotherapy Unit, Ospedale “Vito Fazzi”, Lecce, Italy; 15 Radiation Oncology, IRCCS Regina Elena National Cancer Institute, Rome, Italy; 16 Radiotherapy Unit, Cannizzaro Hospital, Catania, Italy; 17 Radiation Oncology Unit - Department of Biomedical, Dental Science and Morphological and Functional Images, University of Messina, Messina, Italy; 18 Radiation Oncology Unit of Arezzo-Valdarno, Azienda USL Toscana Sud Est, Arezzo, Italy; 19 Department of Radiation Oncology, Policlinico San Matteo Pavia Fondazione IRCCS, Pavia, Italy

**Keywords:** Localized prostate cancer, Volumetric image-guided radiotherapy, Outcome, Toxicity

## Abstract

**Background:**

Moderate hypofractionated radiotherapy is a treatment option for the cure of localized prostate cancer (PCa) patients based on the results of randomized prospective trials, but there is a clinical concern about the relatively short length of follow-up, and real-world results on outcome and toxicity based on cutting-edge techniques are lacking. The objective of this study is to present the long-term results of a large multicentric series.

**Materials and methods:**

We retrospectively evaluated 1325 PCa patients treated with daily volumetric image-guided hypofractionated radiotherapy between 2007 and 2020 in 16 Centers. For survival endpoints, we used Kaplan–Meier survival curves and fitted univariate and multivariable Cox’s proportional hazards regression models to study the association between the clinical variables and each survival type.

**Results:**

At the end of the follow-up, 11 patients died from PCa. The 15-year values of cancer-specific survival (CSS) and biochemical relapse-free survival (b-RFS) were 98.5% (95%CI 97.3–99.6%) and 85.5% (95%CI 81.9–89.4%), respectively. The multivariate analysis showed that baseline PSA, Gleason score, and the use of androgen deprivation therapy were significant variables for all the outcomes. Acute gastrointestinal (GI) and genitourinary (GU) toxicities of grade ≥ 2 were 7.0% and 16.98%, respectively. The 15-year late grade ≥ 2 GI and GU toxicities were 5% (95%CI 4–6%) and 6% (95%CI 4–8%), respectively.

**Conclusion:**

Real-world long-term results of this multicentric study on cutting-edge techniques for the cure of localized PCa demonstrated an excellent biochemical-free survival rate of 85.5% at 15 years, and very low rates of ≥ G3 late GU and GI toxicity (1.6% and 0.9% respectively), strengthening the results of the available published trials.

## Introduction

External beam radiotherapy (EBRT) is a standard treatment option for the cure of localized prostate cancer (PCa) [1, 2]. Based on differences in terms of radiosensitivity of the irradiated tissues (e.g., prostate tumor, rectum, bladder) [3], which is characterized by the α/β ratio, during the last 15 years several prospective trials have been developed to compare conventional fractionation (74–80 Gy delivered as 37–40 fractions of 2 Gy, five fractions per week) with moderate hypofractionation (2.5–3.5 Gy daily fractions, five fractions per week, total dose of 60–72 Gy). The largest three, PROFIT, CHHiP, and NRG Oncology 0415 demonstrated the non-inferiority of moderate hypofractionation in terms of outcomes and toxicity [4–6]. Despite the results of these studies, there is a clinical concern regarding the relatively short length of follow-up [1, 2]. Moreover, data on cutting-edge techniques in moderate hypofractionated radiotherapy are lacking. For instance, portal imaging at weekly intervals was used to verify treatment accuracy in the CHHiP trial [5], whereas in the PROFIT [4] and NRG Oncology 0415 trial [6], intensity-modulated radiotherapy (IMRT) was not mandatory.

Here, we present the long-term analysis of toxicity and survival of a large multicentric retrospective study on moderate hypofractionated radiotherapy in localized PCa (IPOPROMISE) with daily volumetric image-guidance and intensity-modulated (IMRT) or volumetric modulated arc therapy (VMAT).

## Patients and methods

We retrospectively collected data from 1325 clinically localized PCa patients treated with moderately image-guided hypofractionated EBRT in 16 Italian Centers between 2007 and 2020. Inclusion criteria were age > 18 yr; biopsy-proven adenocarcinoma of the prostate; Eastern Oncology Cooperative Group (ECOG) 0–1; staging computed tomography (CT) scans and/or bone scans for unfavorable-intermediate and high-risk disease. All patients provided informed consent for this analysis. The protocol [Image-guided moderately hyPO-fractionated radiotherapy for localized prostate cancer. A multicentric retrospective study (IPOPROMISE)] was approved by Ethical Committee—Regione Umbria (Approval N. 25991/22/ON, 26/10/2022).

### Treatment

Prostate EBRT consisted of moderate hypofractionation (2.5–3.1 Gy per fraction, total dose of 60–72.8 Gy) and daily volumetric image guidance. The clinical target volume (CTV) included the prostate only for patients with low-risk disease and the prostate and proximal seminal vesicles (at least 1 cm) for those with intermediate- or high-risk PCa. The planning target volume (PTV) encompassed the clinical target volume with anisotropic margins of 4–8 mm. Rectum, bladder, penile bulb, and femurs were defined as organs at risk (OARs) on planning CT. Image-guided radiotherapy (IGRT) was Linac-based with daily cone-beam CT (CBCT) in 1122 (84.6%) patients and tomotherapy-based with daily Megavolt-CT (MVCT) in 203 (15.4%).

### Follow-up and statistics

The follow-up schedule, starting from the end of radiotherapy, consisted of clinical and biochemical evaluation every 3 months during the first 2 years and then every 6 months. Biochemical recurrence was defined as a rise in PSA by 2 ng/ml or more above the nadir PSA (Phoenix definition) [7]. At biochemical recurrence, metastatic disease was defined as any image- or histologically-based diagnosis of PCa outside of the prostate. Cancer-specific mortality was defined as death directly related to PCa progression. Overall survival (OS), metastasis-free survival (MFS), cancer-specific survival (CSS), and biochemical relapse-free survival (b-RFS) were calculated from the end date of radiotherapy to the last follow-up. Toxicity was registered according to the Common Terminology Criteria for Adverse Events (CTCAE) v4.03. Acute (within 90 days from the start of radiotherapy) and late toxicity (>90 days from the start of radiotherapy) were registered.

For each of the four survival endpoints (OS, CSS, MFS, and b-RFS), we applied the Chi-square test to compare the distribution of several patients, tumor, and treatment-related variables, among patients who experienced versus those who did not experience the corresponding event of interest. We then used Kaplan–Meier survival curves (with log-rank test) and fitted univariate and multivariable Cox’s proportional hazards regression models to study the association between the aforementioned variables and each survival type. All statistical tests were two-sided and a p-value was considered significant when lower than 0.05.

## Results

Patient and treatment features are reported in Table 1. The median age of PCa diagnosis was 74.6 years (interquartile [IQR], 70.8–77.6 years), the median PSA was 7.93 ng/ml (IQR, 5.78–12 ng/ml), and 68.5% of the patients had a T1–T2 disease. The Gleason score was ≥ 8 in 278 (21%) patients. Based on NCCN risk grouping, 373 (28.1%) patients had low-risk, 492 (37.3%) intermediate risk, 419 (31.6%) high-risk, and 41 (3%) very high-risk disease. The median EBRT total dose was 70.2 Gy (IQR, 62–70.2 Gy), and only 5.1% were treated with 3DCRT (Table 1), whereas all the others received IMRT (27.6%) or VMAT (67.3%). At the time of EBRT, 698 (52.7%) of the patients were on androgen deprivation therapy (ADT).

**Table 1:** Patient (no 1325) and treatment features

Parameter	Result
*Age at diagnosis (yr)*	
Median (IQR)	74.65(70.77–77.60)
*Initial PSA (ng/ml)*	
Median (IQR)	7.93 (5.78–12.00)
*Clinical stage, no (%)*	
T1	211 (16)
T2	695 (52.5)
T3	274 (20.7)
T4	5 (0.3)
Not reported	140 (10.5)
*Biopsy Gleason score, no (%)*	
6	461 (34.8)
7	586 (44.2)
8	165 (12.5)
9	89 (6.7)
10	8 (0.6)
Not reported	16 (1.2)
*Risk group, no (%)*	
Low	373 (28.1)
Favorable intermediate	289 (22)
Unfavorable intermediate	203 (15.3)
High	419 (31.6)
Very high	41 (3)
*ISUP grading, no (%)*	
1	463 (34.9)
2	357 (26.9)
3	228 (17.2)
4	165 (12.5)
5	96 (7.2)
Not reported	16 (1.2)
*ADT, no (%)*	
No	627 (47.3%)
Yes	698 (52.7%)
*Patients in ADT based on risk class, no (%)*	
Low (no = 373)	75 (20.1)
Favorable intermediate (no = 289)	148 (51.2)
Unfavorable intermediate (no = 203)	128 (63)
High (no = 419)	308 (73.5)
Very high (no = 41)	39 (95.1)
*Radiotherapy total dose (Gy)*	
Median (IQR)	70.2 (62–70.2)
*Radiotherapy technique, no (%)*	
3DCRT	68 (5.1)
IMRT	365 (27.6)
VMAT	892 (67.3)

Yr, years; PSA, prostate-specific antigen; ISUP, International Society of Urological Pathology; ADT, androgen deprivation therapy; 3DCRT, three-dimensional conformal radiation therapy; IMRT, intensity modulated radiation therapy; VMAT, volumetric modulated arc therapy

**Fig. 1:**
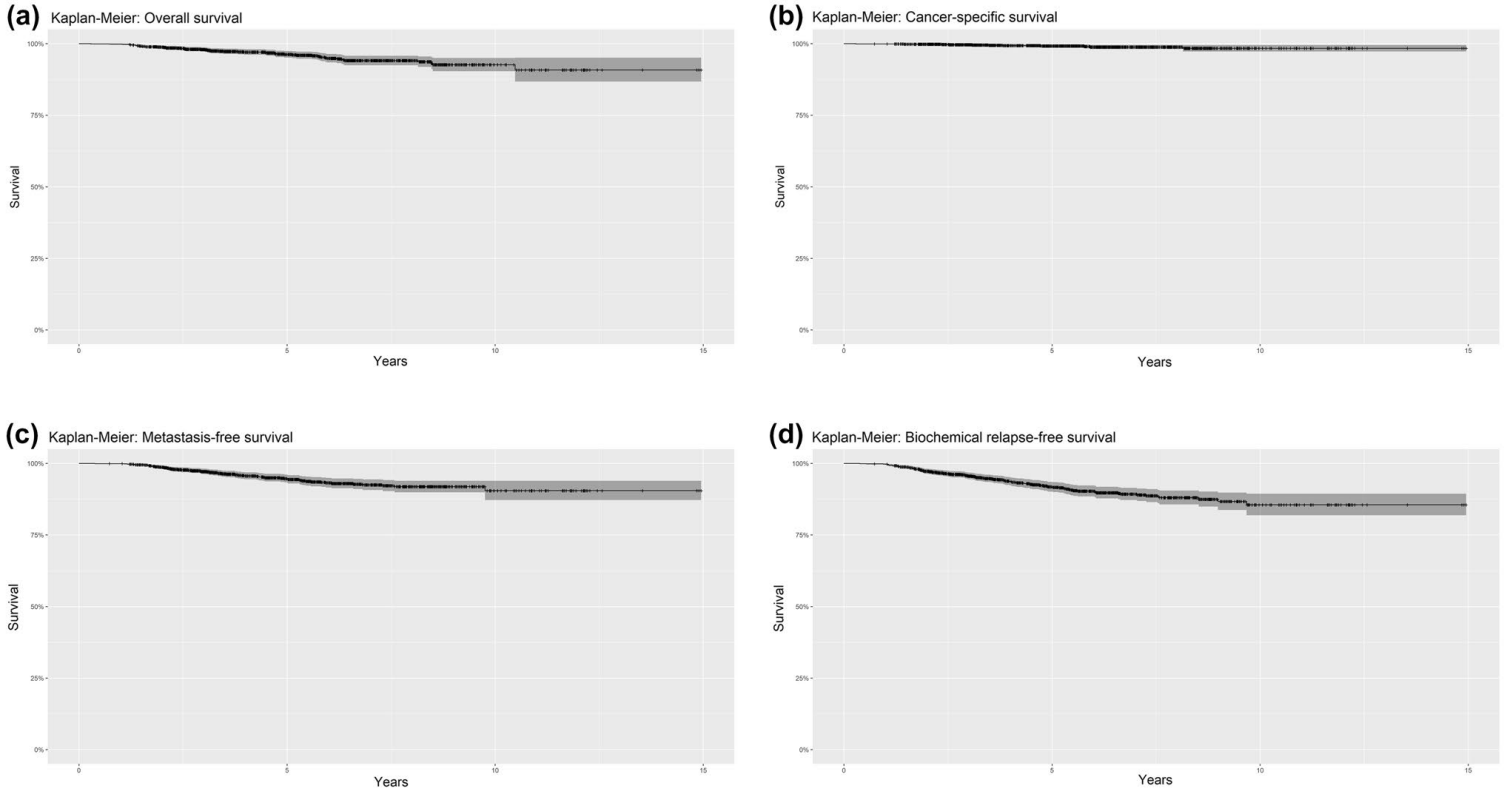
Survival curves. **a** Overall survival (OS). **b** Cancer-specific survival (CSS). **c** Metastasis-free survival (MFS). **d** Biochemical relapse-free survival (b-RFS)

The median follow-up was 5.2 years (IQR 3.2–7.5). A total of 57 patients died (from any cause) during follow-up: the OS was 92.6% (95%CI 90.3–95.0%) and 90.8% (95%CI 86.7–95.1%), at 10 and 15 years of follow-up, respectively (Fig. 1a), and the median OS was 17.3 years (IQR 17.3—not reached). Only 11 patients died from prostate cancer during follow-up: the median CSS was not reached, and the 10- and 15-year valueswere both 98.5% (95%CI 97.3–99.6%) (Fig. 1b). Distant metastases were detected during follow-up among 71 patients: the median MFS was not reached, and the rate of MFS was 90.5% both at 10 and at 15 years (95%CI 87.2–93.9%) (Fig. 1c). Finally, b-RFS (median 17.1 years, IQR, 17.1—not reached) was 85.5% (95%CI 81.9–89.4%) at 10 and 15 years, with a total of 107 patients experiencing a biochemical relapse during follow-up (Fig. 1d).

**Fig. 2:**
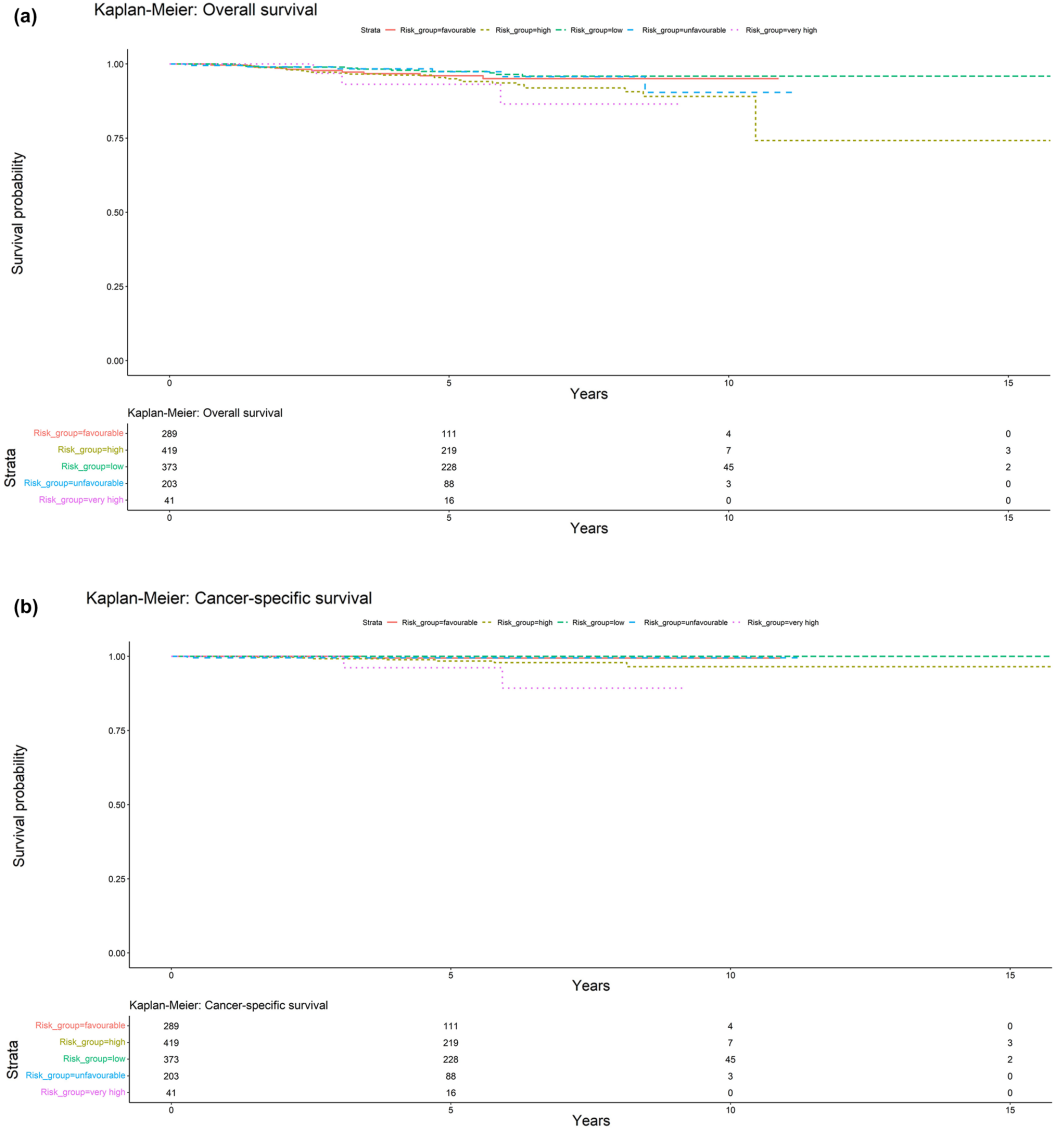

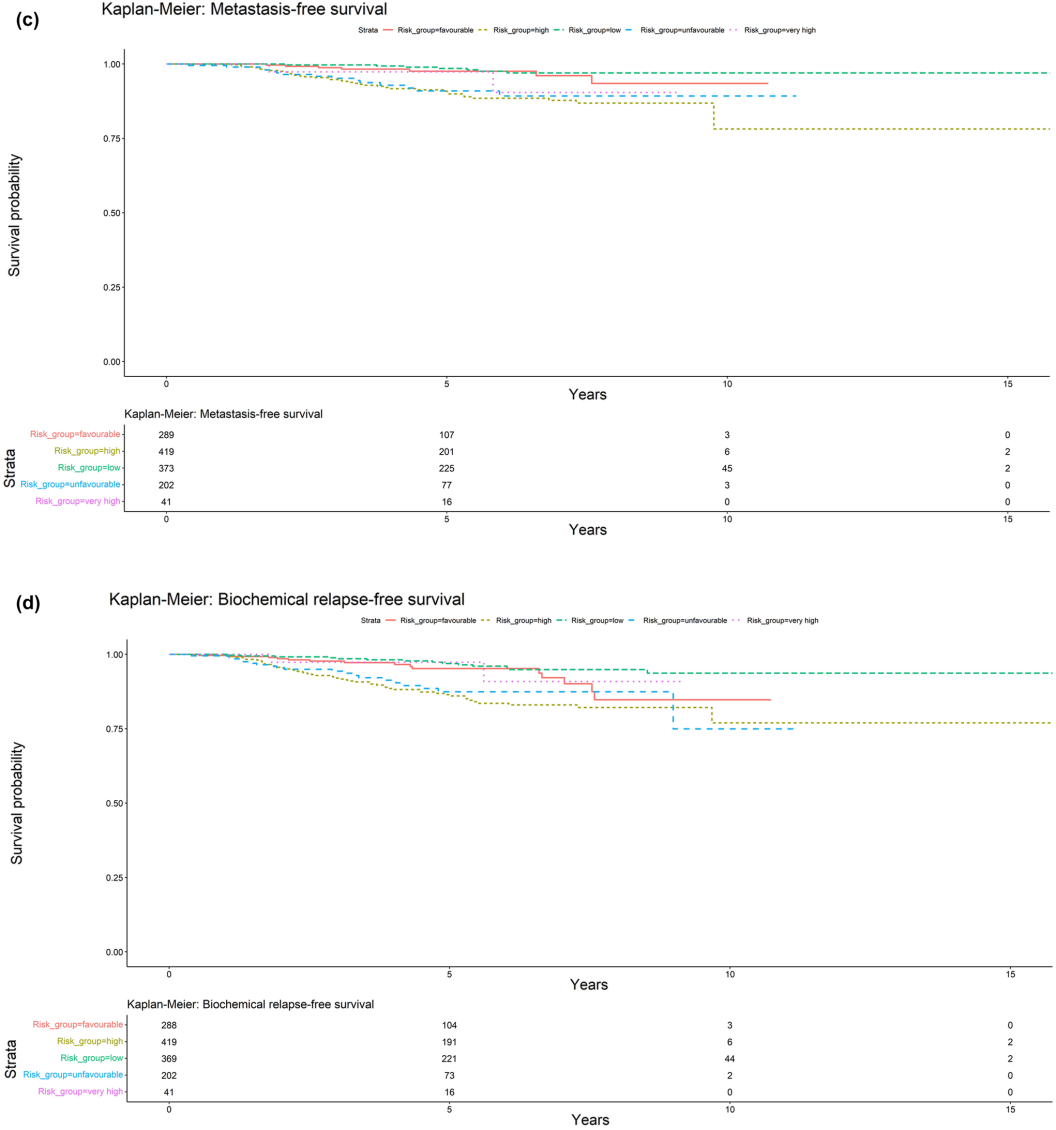
Kaplan–Meier curves stratified by risk class (low: green; favorable-intermediate: red; unfavorable-intermediate: light blue; high: yellow-green; very-high: purple). **a** Overall survival (OS). **b** Cancer-specific survival (CSS). **c** Metastasis-free survival (MFS). **d** biochemical relapse-free survival (b-RFS)

The Kaplan–Meier curves for the entire cohort and stratified by risk group are reported in Fig. 2. All the survival curves show a behavior concordant to their respective risk classes, associating higher risk classes to lower survival rates (with minor differences most likely attributable to the limited number of patients in each risk class). Moreover, the p-value from the log-rank test was below 0.05 for all the analyses, thus confirming that patients’ survival significantly differed across risk classes.

On univariate analysis, increasing baseline PSA, higher Gleason score, increasing risk class, and increasing International Society of Urological Pathology (ISUP) grade were associated with worse b-RFS, MFS, and OS (Tables 2, 3). The multivariate analysis confirmed that baseline PSA and Gleason score were significant variables for all the outcomes. Regarding the use of ADT, in both univariate and multivariate analysis there was an association with worse oncologic outcomes (Tables 2, 3).

**Table 2:** Univariate and multivariate logistic regression analysis for biochemical-free survival and metastasis-free survival (1325 patients)

	biochemical-free survival					Metastasis-free survival				
Variable	log-rank test	Univariate Cox regression		Multivariate Cox regression		log-rank	Univariate Cox regression		Multivariate Cox regression	
	p-value	HR (95%CI)	p-value	HR (95%CI)	p-value	p-value	HR (95%CI)	p-value	HR (95%CI)	p-value
*Age at diagnosis (yr)*										
≤ 69.5	0.742	1.00		1.00		0.847	1.00		1.00	
> 69.5 and ≤ 73.3		0.88 (0.50–1.55)	0.662	0.89 (0.50–1.59)	0.693		1.12 (0.54–2.33)	0.751	1.07 (0.51–2.25)	0.856
> 73.3 and ≤ 75.9		0.94 (0.54–1.65)	0.837	0.88 (0.49–1.56)	0.657		1.21 (0.59–2.49)	0.595	1.14 (0.55–2.35)	0.721
> 75.9 and ≤ 78.2		0.65 (0.35–1.22)	0.183	0.56 (0.29–1.08)	0.082		0.83 (0.38–1.83)	0.649	0.68 (0.30–1.56)	0.368
> 78.2		0.90 (0.50–1.64)	0.744	0.70 (0.37–1.32)	0.272		1.25 (0.59–2.62)	0.558	1.14 (0.53–2.44)	0.736
*Initial PSA (ng/mL)*										
< 10	< 0.001*	1.00		1.00		< 0.001*	1.00		1.00	
≥ 10 and < 20		2.08 (1.33–3.25)	0.001*	1.56 (0.97–2.50)	0.063		2.15 (1.25–3.72)	0.006*	1.65 (0.94–2.90)	0.079
> 20		3.17 (1.94–5.20)	< 0.001*	1.70 (0.98–2.95)	0.060		3.05 (1.64–5.65)	< 0.001*	1.90 (0.99–3.64)	0.054*
*Tumor stage*										
T1	0.216	1.00				0.147	1.00			
T2		1.53 (0.82–2.85)	0.185				1.37 (0.63–2.97)	0.419		
T3-T4		1.81 (0.92–3.55)	0.083				2.04 (0.91–4.57)	0.082		
*Gleason score*										
6	< 0.001*	1.00		1.00		< 0.001	1.00			
7		2.92 (1.70–5.03)	< 0.001*	2.47 (1.38–4.41)	0.002*		3.87 (1.84–8.14)	< 0.001*		
8		4.21 (2.20–8.07)	< 0.001*	3.11 (1.53–6.34)	0.002*		5.17 (2.16–12.36)	< 0.001*		
9–10		6.26 (3.18–12.34)	< 0.001*	4.17 (1.95–8.92)	< 0.001*		11.01 (4.74–25.57)	< 0.001*		
*Risk class*										
Low		1.00					1.00			
Favorable intermediate	< 0.001*	1.83 (0.88–3.81)	0.105			< 0.001*	1.70 (0.59–4.88)	0.320		
Unfavorable intermediate		3.50 (1.76–6.95)	< 0.001*				5.17 (2.10–12.74)	< 0.001*		
High		4.23 (2.35–7.62)	< 0.001*				5.91 (2.63–13.26)	< 0.001*		
Very high		1.73 (0.39–7.65)	0.467				3.53 (0.73–17.04)	0.117		
*ISUP grading*										
1	< 0.001*	1.00				< 0.001*	1.00			
2		2.29 (1.26–4.15)	0.006*				2.35 (1.04–5.31)	0.04*		
3		3.58 (1.95–6.57)	< 0.001*				5.37 (2.49–11.62)	< 0.001*		
4		3.77 (1.96–7.22)	< 0.001*				4.24 (1.79–10.04)	0.001*		
5		5.98 (3.06–11.69)	< 0.001*				9.97 (4.41–22.56)	< 0.001*		
*ADT*										
No		1.00		1.00			1.00		1.00	
Yes	< 0.001*	2.57 (1.69–3.91)	< 0.001*	1.56 (0.96–2.52)	0.07	< 0.001*	3.55 (2.03–6.22)	< 0.001*	3.09 (1.68–5.65)	< 0.001*

HR, hazard ratio; CI, confidence interval; yr, years; PSA, prostate-specific antigen; ISUP, International Society of Urological Pathology; ADT, androgen deprivation therapy

*Significant value

**Table 3:** Univariate and multivariate logistic regression analysis for overall survival (1325 patients)

Variable	*Overall survival*				
	log-rank test	Univariate Cox regression		Multivariate Cox regression	
	p-value	HR (95%CI)	p-value	HR (95%CI)	p-value
*Age at diagnosis (yr)*					
≤ 69.5	0.62	1.00		1.00	
> 69.5 and ≤ 73.3		0.96 (0.41–2.26)	0.921	0.83 (0.34–2.03)	0.688
> 73.3 and ≤ 75.9		1.06 (0.46–2.45)	0.890	0.98 (0.42–2.28)	0.967
> 75.9 and ≤ 78.2		1.00 (0.42–2.37)	0.994	0.78 (0.32–1.91)	0.590
> 78.2		1.65 (0.74–3.64)	0.218	1.29 (0.56–2.96)	0.549
*Initial PSA (ng/mL)*					
< 10	< 0.004*	1.00		1.00	
≥ 10 and < 20		2.48 (1.39–4.41)	0.002*	1.96 (1.06–3.61)	0.031*
> 20		2.02 (0.94–4.36)	0.073	1.38 (0.60–3.17)	0.454
*Tumor stage*					
T1	0.114	1.00			
T2		1.26 (0.55–2.93)	0.584		
T3-T4		2.13 (0.89–5.09)	0.090		
*Gleason score*					
6	0.013*	1.00		1.00	
7		1.95 (1.01–3.77)	0.046*	1.31 (0.66–2.63)	0.438
8		1.67 (0.64–4.38)	0.298	0.97 (0.35–2.68)	0.954
9–10		3.99 (1.66–9.60)	0.002*	2.26 (0.86–5.93)	0.098
*Risk class*					
Low	0.050*	1.00			
Favorable intermediate		1.35 (0.49–3.67)	0.560		
Unfavorable intermediate		1.65 (0.69–3.93)	0.257		
High		2.55 (1.26–5.19)	0.009*		
Very high		3.61 (1.00–13.07)	0.050*		
*ISUP grading*					
1	0.036*	1.00			
2		1.92 (0.95–3.87)	0.069		
3		1.48 (0.62–3.51)	0.377		
4		1.56 (0.60–4.07)	0.358		
5		3.75 (1.58–8.91)	0.003*		
*ADT*					
No		1.00		1.00	
Yes	0.002*	2.38 (1.35–4.22)	0.003*	1.96 (1.01–3.81)	0.047*

HR, hazard ratio; CI, confidence interval; yr, years; PSA, prostate-specific antigen; ISUP, International Society of Urological Pathology; ADT, androgen deprivation therapy

*Significant value

The toxicity analysis showed that only 6 patients (0.45%) developed acute gastrointestinal (GI) grade 3 (G3) toxicity (consisting of rectal bleeding [1 patient] and proctitis [5 patients]), and 93 (7.0%) patients a grade 2. Acute genitourinary (GU) toxicity of grade ≥ 2 was registered in 225 (16.98%), with only 12 (0.9%) having a G3 (consisting of hematuria [3 patient], cystitis [8 patients], and urinary obstruction [1 patient]). No patient experienced grade 4 GI and/or GU acute toxicity.

**Fig. 3:**
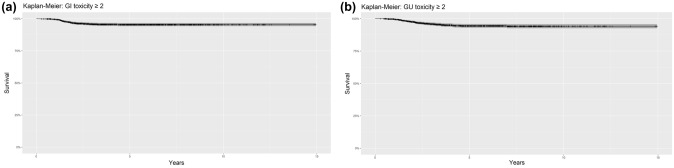
Late toxicity. **a** Late grade ≥ 2 GI toxicity. **b** Late grade ≥ 2 GU toxicity

Late GI toxicity of grade ≥ 2 was registered in 54 (4%) patients, with 12 (0.9%) having a G3 (rectorrhagia 8, proctitis 4). The 5-, 10- and 15-year late grade ≥ 2 GI toxicity was 5% (95%CI, 4–6%) since no patient developed high-grade GI toxicity between 5 and 15 years of follow-up (Fig. 3a).

Late GU toxicity of grade ≥ 2 was registered in 61 (4.6%), with 21 (1.6%) having a G3 (consisting of hematuria [5 patients], cystitis [4 patients], and urinary obstruction [12 patients]). The 5-year late grade ≥ 2 GU toxicity was 5% (95%CI, 4–7%), whereas the rate was 6% (95%CI, 4–8%) at 10 and 15 years since no patient developed late grade  ≥ 2 GU toxicity between 10 and 15 years of follow-up (Fig. 3b).

## Discussion

Moderate hypofractionation (between 2.5 and 3.5 Gy per fraction) is a standard treatment option for patients affected by localized PCa based on several randomized controlled trials (RCTs) involving up to 3000 patients, with median follow-up ranging from 5 to 10 years, who received mainly three-dimensional conformal radiation therapy (3DCRT) without daily volumetric image guidance [4, 5, 8]. Compared with 3DCRT, cutting-edge treatment planning, and delivery technologies have progressively been used in clinical practice, allowing hypofractionation and minimizing the risk of toxicity [9]. To our knowledge, here we have reported the largest multicentric real-world series of daily volumetric image-guided moderately hypofractionated radiotherapy (IMRT and VMAT based) for localized PCa, with a median follow-up time of over 5 years. Our study adds information about the safety and efficacy of this treatment option for the cure of localized PCa patients and highly supports the use of this treatment for any risk class of the disease. Taking into account that about 50% of the patients were in the highest risk categories, the most interesting results are the very low rate of CSS at 10 and 15 years with only 11 (0.8%) patients dead from PCa at the end of follow-up, and only 5.3% of the total cohort experiencing a metastatic disease. Analyzing the clinical features affecting outcomes, higher baseline PSA, higher Gleason score, and the use of ADT were all associated with worse survival and these results at multivariable analysis (Tables 2, 3) are consistent with those from other studies such as the one of Abu-Gheida et al. [10] who recently reported the 10-year analysis of a large mono-institutional series of localized PCa treated with moderately hypofractionated IMRT. Long-term data from Abu-Gheida et al. [10] on grade ≥ 3 GU and GI toxicity evidenced a 10-year cumulative incidence rate of 2% and 1%, respectively. Accordingly, in our series, the rate of late grade ≥ 3 toxicity at the end of follow-up was 1.6% for GU and 0.9% for GI, with no patient experiencing a G4 late toxicity.

Recently at the ASCO GU 2023 symposium, the 10-year results of the CHHiP trial were presented confirming the non-inferiority of the hypofractionation arm (60 Gy/20 fractions) (HR = 0.84, 95%CI 0.72, 0.97) and a borderline significance for superiority (HR = 0.84, 95%CI 0.70, 1.00). The efficacy of this treatment schedule was confirmed in terms of biochemical failure-free survival (79.8%, 95%CI 77.1–82.3), distant metastases-free survival (94.3%, 95%CI 92.7–95.6), and overall survival (83%, 95%CI 80.5–81.5), with low rates of bladder and rectal toxicity [11].

Several randomized trials comparing conventional fractionation with moderate hypofractionation did not show a significant difference in terms of toxicity [4, 5, 8, 12], although some others reported higher acute and late toxicity in the hypofractionation arm [6, 13]. In the range of dose per fraction and total dose of moderate hypofractionation, the biologically effective dose (BED) could help explain why there is evidence in some of the randomized trials [6, 13] of increased late effects. To reduce the risk of late toxicity compared with conventional fractionation, for an α/β ratio ranging from 1.5 to 2.5 Gy, it has been hypothesized that BEDs for moderate hypofractionation should not exceed a BED1.5Gy of 183 Gy 1.5 Gy or a BED2.5Gy of 136 Gy2.5Gy [14]. This means that the radiation therapy schedule should be comprised between 27 x 2.5 Gy (total dose, 67.5 Gy) and 17 × 3.3 Gy (total dose, 56.1 Gy). In the present series, the median total dose of 70.2 Gy (26 × 2.7 Gy) corresponds to 196.5 Gy1.5Gy which is above the set limit proposed by Brenner and Hall, but if we consider the toxicity data we had a very low rate of grade 3 (0.9% for GI and 1.6% for GU) and a 5-year late grade ≥ 2 GI and GU toxicities of 5%, respectively. For instance, in the NRG Oncology RTOG-0415 trial [6], where a total dose of 70 Gy (28 × 2.5 Gy, corresponding to 186.7 Gy1.5Gy) was delivered to 545 patients, the rate of late grade 2 GI and GU were 18.3% and 26.2%, with G3 late toxicity of 4.1% for GI and 3.5% for GU. To explain our favorable toxicity results despite the high BED of 196.5 Gy1.5Gy, we have to consider that we collected data from patients treated with modern techniques (IMRT and VMAT) with daily volumetric image guidance allowing smaller CTV to PTV expansion. The availability of cutting-edge image-guided high-conformality treatments allows the safe and effective delivery of moderate hypofractionated radiotherapy in clinical practice, and it is leading to the use of stereotactic body radiotherapy (SBRT) as an option for the cure of patients affected by localized PCa [15]. Even though SBRT (1 -5 total fractions with a dose per fraction ≥ 5 Gy) is becoming more and more administered for the treatment of localized PCa, moderate hypofractionation is currently the standard treatment option due to the optimal long-term outcome and toxicity results. The results of the ongoing PACE-C trial, a randomized study directly comparing SBRT and moderate hypofractionated RT, and many more other trials, are eagerly awaited.

The strengths of our study are the real-world data on a very large number of patients with long-term follow-up and the multicentric nature of the study.

## Conclusions

Long-term results of the current study on 1325 PCa patients treated with moderate hypofractionated radiotherapy with daily volumetric image-guidance and IMRT or VMAT technique demonstrated excellent biochemical-free survival rate of 85.5% at 10 and 15 years, and very low rates of ≥ G3 late GU and GI toxicity (1.6 % and 0.9 %, respectively), strengthening the results of the available published RCTs.
